# The need for an organoid manufacturing, preservation, and distribution center

**DOI:** 10.1093/stcltm/szaf031

**Published:** 2025-07-08

**Authors:** Anthony Atala, John C Bischof, Christopher S Chen, John P Fisher, David L Hermanson, Charles L Howe, Walter C Low, Zhen Ma, David H McKenna, Sean P Palecek, Johnna S Temenoff, Brenda M Ogle

**Affiliations:** Wake Forest Institute for Regenerative Medicine, Wake Forest University, Winston-Salem, NC 27101, United States; Advanced Technologies for the Preservation of Biological Systems, NSF ERC, University of Minnesota, Minneapolis, MN 55455, United States; Department of Mechanical Engineering, University of Minnesota, Minneapolis, MN 55455, United States; Cellular Metamaterials, NSF ERC, Boston University, Boston, MA 02215, United States; Department of Biomedical Engineering, Boston University, Boston, MA 02215, United States; The Wyss Institute for Biologically Inspired Engineering, Harvard University, Boston, MA 02115, United States; Fischell Department of Bioengineering, University of Maryland, College Park, MD 20742, United States; Cell and Gene Therapy Applications, Bio-Techne, Minneapolis, MN 55413, United States; Research Office of Core Shared Services, Mayo Clinic, Rochester, MN 55905, United States; Division of Experimental Neurology, Mayo Clinic, Rochester, MN 55905, United States; Stem Cell Institute, University of Minnesota, University of Minnesota, Minneapolis, MN 55455, United States; Department of Neurosurgery, University of Minnesota, Minneapolis, MN 55455, United States; Department of Biomedical and Chemical Engineering, Syracuse University, Syracuse, NY 13244, United States; Molecular and Cellular Therapeutics, University of Minnesota, St. Paul, MN 55108, United States; Department of Laboratory Medicine and Pathology, University of Minnesota, Minneapolis, MN 55455, United States; Cell Manufacturing Technologies, NSF ERC, University of Wisconsin, Madison, WI 53706, United States; Chemical and Biological Engineering, University of Wisconsin, Madison, WI 53706, United States; Cell Manufacturing Technologies, NSF ERC, Georgia Institute of Technology, Atlanta, GA 30332, United States; Department of Biomedical Engineering, Georgia Institute of Technology, Atlanta, GA 30332, United States; Department of Biomedical Engineering, University of Minnesota, Minneapolis, MN 55455, United States

**Keywords:** organoid, biomanufacturing, standardization, cryopreservation, distribution

## Abstract

Organoids, which are tiny, lab-grown 3D structures that mimic some organizational and functional properties of human organs, are slowly transforming the face of systems and developmental biology, biomedical research, pharmaceutical testing, environmental toxin testing, and healthcare. Significant investments are essential for the mass production, preservation, and distribution of organoids, with the aim to accelerate innovation and progress across multiple fields—much like the investments made in cell and biologics manufacturing over the past 2 decades.

## Introduction

Organoids, which are miniaturized and simplified versions of organs grown in vitro from cells, are beginning to make a significant impact on systems and developmental biology, biomedical research, and healthcare. These 3-dimensional structures largely mimic the complexity of real organs, providing unprecedented opportunities for studying basic human and animal biology, disease mechanisms, drug-tissue interactions, environmental toxin-tissue interactions, regenerative therapies, and even adaptive reservoir computation. However, the potential of organoids is constrained by the lack of large-scale organoid manufacturing, preservation, and distribution facilities. Establishing such facilities is crucial for advancing biological research, improving healthcare outcomes, and maintaining leadership in biotechnology.

## Obstacles to organoid production

Several review articles from the past 2 years summarize the exciting advances in organoid development, manufacturing, and use.^1-15^ Organoids representing over 30 human organs or systems can now be produced consistently, including disease-state models of cancer. Many of these have either revealed key biological and developmental processes or have been used for pharmaceutical testing. Many are also being developed as potential regenerative therapies that will overturn the “no treatment possible” paradigms for many diseases. In parallel with these advances, significant developments in sensors, stem cell engineering, Artificial Intelligence (AI)-driven analysis of organoid imaging, and various other ancillary technologies are promoting organoid production and evaluation. Over 20 facilities worldwide (including 8 in the United States) regularly produce organoids, and hundreds of academic labs develop and produce organoids for internal research.

Yet the field is currently constrained to incremental advances for the following reasons:

- Organoids are typically made in small batches. Even the “organoid factories” around the world focus on only a few organoids each, without consistent quality control standards between facilities and in batches too small to conduct large-scale research^11^ (such as high-throughput screening for drug discovery or personalized medicine that may require large numbers of distinct organoid “individuals”).
- Organoid production is labor- and time-intensive in research labs. Many organoids are generated from stem cells, and the process of differentiating a stem cell to somatic cell types currently takes weeks to months in a research lab, even with daily maintenance. Developing organoids generally takes an additional 2-3 weeks.
- Organoids often rely on biologic starting material of varying quality. Some organoids are generated using extracellular matrix proteins that are purified from native tissues and vary substantially in terms of purity and quality from batch to batch.^16,17^ Many organoids are generated from human-induced pluripotent stem cells (hiPSCs), but the number of hiPSC lines produced under cGMP-compliant conditions and available for publicly funded research and clinical trials is very limited.
- Organoids have a short shelf life and are difficult to share. Organoids degrade quickly if removed from narrow physiologic conditions, even at cooler temperatures. Thus, it is costly to maintain organoids at the site of production and very difficult to move them to another location.
- There are limited standards for organoid production and quality control.^3^ Organoids from one lab can differ significantly from those of another, even if grown from the same stem cell line via the same protocol. In addition, structural and functional success criteria are often undefined, making it difficult to compare results across different research labs and companies.

## The next step: biofabrication facilities dedicated to large-scale organoid production, preservation, and distribution

**Figure 1.**
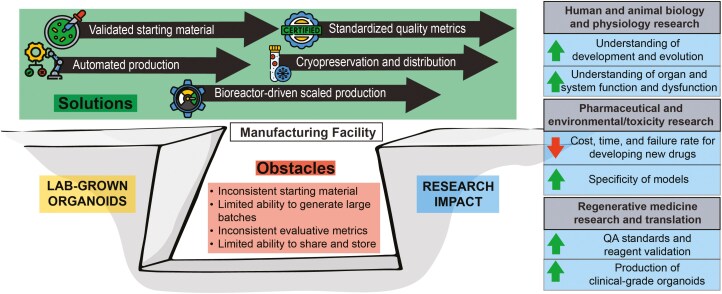
**Bridging the gap from lab-grown organoids to large-scale organoid production via organoid manufacturing, preservation, and distribution centers.** Well-established and nascent technologies are now available to address obstacles barring the full realization of the tremendous research and clinical potential of organoids.

To address these obstacles to the widespread use of organoids, facilities dedicated to large-scale production, quality assurance standardization, preservation, and distribution of organoids are needed (Figure 1). Such facilities should have integrated space for automated production, next-generation bioreactor systems in adjacent cell culture facilities, in-line noninvasive imaging, and analytical data collection, cryopreservation, and storage. The result would be large quantities of organoids available “off-the-shelf,” capable of being safely shipped to any location.

Continued organoid process development is also essential so new organoids can be added to production lines once they can be reliably generated. Gene editing strategies for the creation of organoids displaying mono- or multi-genic disease phenotypes need to be produced in the same manner.

Continued development of organoid cryopreservation is also vital. Without relatively easy cryopreservation protocols at production sites and similarly easy rewarming protocols at labs that receive preserved organoids, there can never be widespread use of these delicate tools. Cryopreservation is routinely used to preserve the precursor cells which ultimately grow together into organoids.^18^ However, to our knowledge there are few examples of stem cell derived, or directly isolated, organoids which have been successfully cryopreserved with high viability and function. Notably, successful cryopreservation and rewarming was achieved for both stem cell-derived beta cell clusters and isolated pancreatic islets in quantities relevant to both research and clinical applications,^19^ with an approach that can likely be modified for other organoids. Other preservation strategies are under development with varied degrees of success depending on the organoid type.^20,21^

Initially, large-scale organoid production facilities should stock the most commonly used organoids for on-demand distribution. The facilities should work with organoid and biopreservation developers and experts to clearly define rigorous production, preservation, and quality control standards and to streamline the process of introducing new organoids to production lines.

The demand for consistent, high-quality organoids is currently high enough to expect production facilities to be self-sufficient within a few years of coming online, both through developing and selling organoids directly and by participating in research grants. Funding for construction and initial operation would likely come from multiple stakeholders, including federal/nonprofit/academic and industry partnerships. A feedforward loop where innovation in one entity will spur and spawn innovation in another would be ideal. For example, many life science companies harbor workflows, reagents, AI-based quality control approaches, biologics distribution, and associated expertise that could augment organoid manufacturing. Simultaneously, these companies often seek access to organoids from qualified and varied sources to validate their medical device or biological products.

Organoids raise ethical and regulatory issues, particularly because they are derived from stem cells and patient biopsy material. Moreover, large-scale production of organoids should be accompanied by availability to labs in smaller companies and R2 and R3 academic institutions in the United States and to researchers throughout middle- and low-income countries. Dedicated centers would be well-positioned to address such issues by developing and adhering to ethical guidelines and working closely with regulatory agencies. We envision this happening on a national level in conjunction with organizations such as the Federation of American Societies for Experimental Biology, the Standards Coordinating Body for Regenerative Medicine, the Federation of State Medical Boards of the United States (FSMB), and the American Federation for Medical Research. At the international level, collaboration with the WHO Expert Committee on Biological Standardization, the International Association of Medical Regulatory Authorities (sponsored by FSMB), and nation-specific regulatory bodies will likewise be essential.

## The future: widespread organoid production and distribution

We envision academic centers, private institutions, and industrial entities either engaging with or building organoid production and preservation facilities at large and small scales. Such a network of facilities would serve to meet a wide variety of research needs in biology and medicine and would bolster local and national economies. We also envision that readily available production facilities will stimulate even more development of organoids themselves.

## References

[1] Groen E , Mummery CL, Yiangou L, Davis RP. Three-dimensional cardiac models: a pre-clinical testing platform. Biochem Soc Trans. 2024;52:1045-1059. https://doi.org/10.1042/BST20230444 38778769 PMC11346450

[2] Imai T , Ishigamori R, Naruse M, et al Bridging toxicological properties of environmental chemicals between animals and humans using healthy organoid systems. J Toxicol Sci. 2024;49:425-434. https://doi.org/10.2131/jts.49.425 39358232

[3] Ahn SJ , Lee S, Kwon D, et al Essential guidelines for manufacturing and application of organoids. Int J Stem Cells. 2024;17:102-112. https://doi.org/10.15283/ijsc24047 38764240 PMC11170116

[4] Yang Y , Cui J, Kong Y, Hou Y, Ma CO. New frontiers in tumor immune microenvironment research. Front Immunol. 2024;15:1422031. 39136020 10.3389/fimmu.2024.1422031 PMC11317300

[5] Jin H , Xue Z, Liu J, et al Advancing organoid engineering for tissue regeneration and biofunctional reconstruction. Biomater Res. 2024;28:0016. https://doi.org/10.34133/bmr.0016 38628309 PMC11018530

[6] Park S , Cho SW. Bioengineering toolkits for potentiating organoid therapeutics. Adv Drug Deliv Rev. 2024;208:115238. https://doi.org/10.1016/j.addr.2024.115238 38447933

[7] Maramraju S , Kowalczewski A, Kaza A, et al Ai-organoid integrated systems for biomedical studies and applications. Bioeng Transl Med. 2024;9:e10641. https://doi.org/10.1002/btm2.10641 38435826 PMC10905559

[8] Wu Y , Ye W, Gao Y, et al Application of organoids in regenerative medicine. Stem Cells. 2023;41:1101-1112. https://doi.org/10.1093/stmcls/sxad072 37724396

[9] Roberto de Barros N , Wang C, Maity S, et al Engineered organoids for biomedical applications. Adv Drug Deliv Rev. 2023;203:115142. https://doi.org/10.1016/j.addr.2023.115142 37967768 PMC10842104

[10] Ashok A , Choudhury D, Fang Y, Hunziker W. Towards manufacturing of human organoids. Biotechnol Adv. 2020;39:107460. https://doi.org/10.1016/j.biotechadv.2019.107460 31626951

[11] Novelli G , Spitalieri P, Murdocca M, Centanini E, Sangiuolo F. Organoid factory: the recent role of the human induced pluripotent stem cells (hipscs) in precision medicine. Front Cell Dev Biol. 2022;10:1059579. https://doi.org/10.3389/fcell.2022.1059579 36699015 PMC9869172

[12] Zhao Z , Chen X, Dowbaj AM, Sljukic A, Bratlie K, Lin L, Fong ELS, Balachander GM, Chen Z, Soragni A, Huch M, Zeng YA, Wang Q, Yu H. Organoids. Nat Rev Methods Primers. 2022;2. https://doi: 10.1038/s43586-022-00174-y PMC10270325 37325195

[13] Cala G , Sina B, De Coppi P, Giobbe GG, Gerli MFM. Primary human organoids models: current progress and key milestones. Front Bioeng Biotechnol. 2023;11:1058970. https://doi.org/10.3389/fbioe.2023.1058970 36959902 PMC10029057

[14] Soto-Gamez A , Gunawan JP, Barazzuol L, Pringle S, Coppes RP. Organoid-based personalized medicine: from tumor outcome prediction to autologous transplantation. Stem Cells. 2024;42:499-508. https://doi.org/10.1093/stmcls/sxae023 38525972 PMC11177156

[15] Verstegen MMA , Coppes RP, Beghin A, et al Clinical applications of human organoids. Nat Med. 2025;31:409-421. https://doi.org/10.1038/s41591-024-03489-3 39901045

[16] Aisenbrey EA , Murphy WL. Synthetic alternatives to matrigel. Nat Rev Mater. 2020;5:539-551. https://doi.org/10.1038/s41578-020-0199-8 32953138 PMC7500703

[17] Heo JH , Kang D, Seo SJ, Jin Y. Engineering the extracellular matrix for organoid culture. Int J Stem Cells. 2022;15:60-69. https://doi.org/10.15283/ijsc21190 35220292 PMC8889330

[18] Matsui K , Sekine H, Ishikawa J, et al Exploration of preservation methods for utilizing porcine fetal-organ-derived cells in regenerative medicine research. Cells. 2024;13:228. https://doi.org/10.3390/cells13030228 38334620 PMC10854901

[19] Zhan L , Rao JS, Sethia N, et al Pancreatic islet cryopreservation by vitrification achieves high viability, function, recovery and clinical scalability for transplantation. Nat Med. 2022;28:798-808. https://doi.org/10.1038/s41591-022-01718-1 35288694 PMC9018423

[20] Han H , Zhan T, Guo N, Cui M, Xu Y. Cryopreservation of organoids: strategies, innovation, and future prospects. Biotechnol J. 2024;19:e2300543. https://doi.org/10.1002/biot.202300543 38403430

[21] Xue W , Li H, Xu J, et al Effective cryopreservation of human brain tissue and neural organoids. Cell *Rep Methods*. 2024;4:100777. https://doi.org/10.1016/j.crmeth.2024.100777 38744289 PMC11133841

